# Ruptured Deep Femoral Artery Aneurysm Caused by Direct Infiltration of Diffuse Large B-Cell Lymphoma

**DOI:** 10.3400/avd.cr.26-00025

**Published:** 2026-06-30

**Authors:** Tomoki Nakatsu, Takayuki Kadohama, Eijiro Hikasa, Hana Fukuta, Yuya Tamaru, Daichi Mizushima, Masahiko Narita, Nobuyuki Akasaka

**Affiliations:** 1 Department of Cardiovascular Surgery, Steel Memorial Muroran Hospital, Muroran, Hokkaido, Japan; 2 Department of Cardiovascular Surgery, Kushiro Kojinkai Memorial Hospital, Kushiro, Hokkaido, Japan

**Keywords:** deep femoral artery aneurysm, diffuse large B-cell lymphoma, rupture

## Abstract

We report a rare case of a ruptured deep femoral artery aneurysm caused by direct infiltration of diffuse large B-cell lymphoma (DLBCL). An 82-year-old man presented with acute left thigh pain and a rapidly formed aneurysm revealed by imaging. Open aneurysmectomy and bypass grafting were performed. Histopathology demonstrated destruction of the elastic laminae and media with diffuse infiltration of atypical lymphoid cells, thus confirming DLBCL. The patient received the best supportive care. Further arterial ruptures developed, and the patient died 8 months after surgery. Malignancy should be considered in the differential diagnosis of rapidly progressive, peripheral aneurysms.

## Introduction

A deep femoral artery aneurysm (DFAA) is a rare type of peripheral arterial aneurysm that is most commonly caused by atherosclerosis, trauma, or infection.^1,2)^ Peripheral arterial aneurysms caused by primary malignant tumors are also rare, and only a few such cases caused by malignant lymphoma have been reported.^3,4)^ We present a case of a ruptured DFAA caused by direct infiltration of diffuse large B-cell lymphoma (DLBCL).

## Case Report

An 82-year-old man who had undergone emergent endovascular aneurysm repair (EVAR) for rupture of the left common iliac artery 1 year previously presented for outpatient follow-up (**Fig. 1A** and **1B**). Intermittent claudication of the left lower extremity was observed, and contrast-enhanced computed tomography (CECT) revealed thrombotic occlusion of the stent graft. Subsequently, he underwent thrombectomy with placement of an additional proximal cuff and bilateral distal limb extensions (**Fig. 1C**). The postoperative course was uneventful, and the patient was discharged on postoperative day 9.

**Fig. 1:**
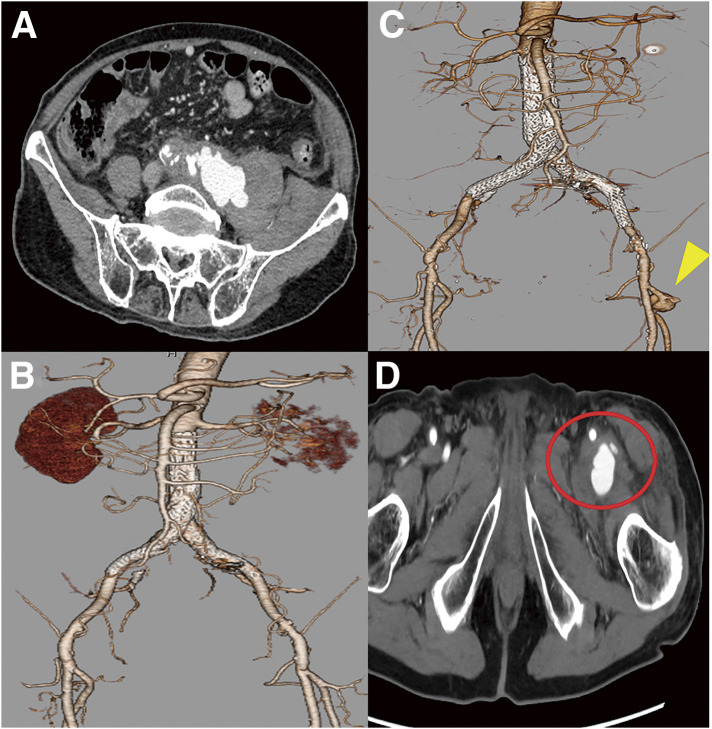
Open aneurysmectomy and graft replacement were performed on an 82-year-old man presenting with acute left thigh pain and a rapidly formed aneurysm. (**A**) CT images obtained at the time of the initial rupture of the left CIAA. (**B**) 3D-CT image obtained after EVAR. (**C**) 3D-CT image obtained at the time of DFAA rupture. The arrow points to a DFAA. (**D**) Axial contrast-enhanced CT image obtained at the time of DFAA rupture. The circle points to a ruptured DFAA. CT: computed tomography; CIAA: common iliac artery aneurysm; DFAA: deep femoral artery aneurysm; EVAR: endovascular aortic repair; 3D: three-dimensional

Two weeks after discharge, the patient returned to the emergency department with acute left thigh pain. A physical examination revealed localized swelling and tenderness of the left thigh without skin erythema or a palpable pulsatile mass. Peripheral pulses were preserved. Laboratory tests revealed mild anemia and a normal white blood cell count; however, the C-reactive protein level was elevated to 5.47 mg/dL. CECT revealed a rapidly formed 25 × 35-mm aneurysm of the left deep femoral artery with a surrounding hematoma and active contrast extravasation (**Fig. 1D**). The patient was diagnosed with a ruptured DFAA.

Because the aneurysm had formed rapidly, the possibility of an infected aneurysm could not be ruled out; therefore, in situ graft replacement was selected instead of endovascular treatment. The femoral arteries were exposed and controlled through a left groin incision. The aneurysm wall appeared whitish and degenerated, with severe adhesions to the surrounding tissues (**Fig. 2A**). The aneurysm was resected, and in situ graft replacement was performed using a great saphenous vein graft (**Fig. 2B**). The total operative time was 179 min, and the estimated blood loss was 1160 mL.

**Fig. 2:**
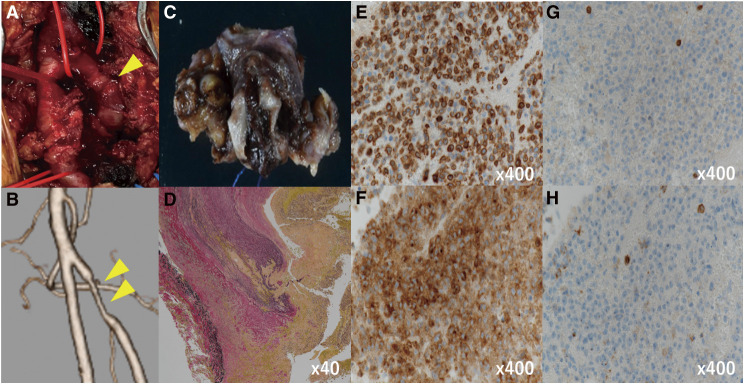
In situ graft replacement exposed the femoral arteries through a left groin incision. (**A**) Intraoperative view of the deep femoral artery aneurysm. The aneurysmal lesion is indicated by an arrowhead. (**B**) Postoperative 3D-CT image obtained after graft replacement. The bypassed segment is indicated by arrowheads. (**C**) Gross appearance of the resected aneurysm specimen (20 × 14 × 12 mm). (**D**) Section stained with hematoxylin and eosin showing extensive tumor cell infiltration. Immunohistochemical staining indicating (**E**) positive for CD79a, (**F**) positive for CD30, (**G**) negative for CD3, and (**H**) negative for CD20. 3D: three-dimensional; CT: computed tomography

Histopathological examination revealed disruption of the internal elastic lamina, media, and external elastic lamina with an associated hemorrhage. Diffuse infiltration of atypical lymphoid cells within the arterial wall was also observed. Immunohistochemical analysis indicated that the cells were positive for CD79a and CD30 and negative for CD3 and CD20, leading to a diagnosis of DLBCL (**Fig. 2C**–**2H**). Positron emission tomography-computed tomography revealed abnormal uptake around the left renal artery and left deep femoral artery, resulting in a diagnosis of stage IV disease (**Fig. 3A**). Systemic chemotherapy was recommended; however, the patient declined treatment and opted for best supportive care. Subsequently, he was transferred to another hospital 2 months after surgery.

**Fig. 3:**
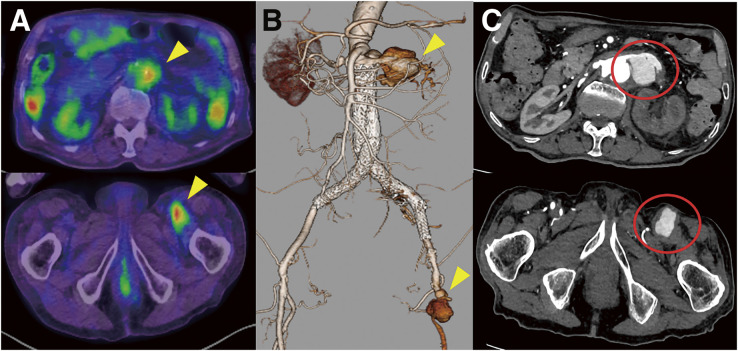
Positron emission tomography-CT revealed abnormal uptake, resulting in a diagnosis of stage IV disease. (**A**) Axial positron emission tomography-CT image demonstrating abnormal uptake in the left renal artery and left deep femoral artery. (**B**) 3D-CT image obtained at the time of repeat rupture. The ruptured sites are indicated by arrowheads. The upper arrow points to a left renal artery aneurysm, whereas the lower arrow points to a DFAA. (**C**) Axial CT image obtained at the time of repeat rupture. The ruptured lesions are indicated by red circles. CT: computed tomography; 3D: three-dimensional; DFAA: deep femoral artery aneurysm

Five months after transfer, the patient reported tenderness in the left thigh. CECT revealed rupture of the left renal artery and left common femoral artery (**Fig. 3B** and **3C**). Because of his poor general condition, re-operation was proposed; however, the patient declined further surgical intervention. Therefore, conservative management was performed. The patient died 8 months after the surgery.

## Discussion

This case represents an extremely rare condition in which DLBCL directly infiltrated the deep femoral arterial wall, resulting in aneurysm formation and rupture. Although a DFAA is difficult to diagnose, determining DLBCL as the cause is even more challenging. Considering the aggressive clinical course and recurrent rupture in the current case, including malignant lymphoma in the differential diagnosis of a DFAA was crucial.

Vascular invasion by malignant lymphoma is rare, and most reported aneurysmal presentations involve the aorta rather than the peripheral arteries.^4)^ To the best of our knowledge, there is only one other report of DLBCL that initially presented as an isolated DFAA with rupture.^5)^ In the present case, the aneurysm expanded rapidly; therefore, the possibility of an infected aneurysm was concerning. In situ graft replacement enabled complete excision of the lesion and provided a specimen for histopathological examination, which was essential for establishing the diagnosis of DLBCL. When an infected aneurysm cannot be ruled out, considering the possibility of malignancy-related aneurysmal disease and selecting open surgery rather than an endovascular approach may be justified, not only from a therapeutic standpoint but also from a diagnostic standpoint.

DLBCL is the most common subtype of non-Hodgkin lymphoma and accounts for approximately 30% of such cases; however, its clinical behavior is heterogeneous. CD20-negative DLBCL is rare, comprising only 1%–2% of all DLBCL cases, and is associated with aggressive progression, treatment resistance, and a poor prognosis.^6)^ The mechanism of aneurysm formation in malignant lymphoma is thought to involve direct infiltration of tumor cells into the arterial wall, leading to destruction of the elastic lamina and media and resulting in the loss of structural integrity.^3,4)^ These findings were consistent with the histopathological features observed in the present case. Local inflammatory responses and cytokine release may further accelerate arterial wall fragility. CD20-negative/CD30-positive DLBCL may behave aggressively, and vascular involvement can lead to rapid progression with recurrent rupture, as observed in the current case.^3)^

Outcomes of lymphoma-associated aneurysms depend on local control of the vascular lesion and systemic treatment of the underlying malignancy.^7)^ In the current case, systemic chemotherapy was not feasible because of the patient’s poor general condition. During the process of diagnosing peripheral arterial lesions, especially in cases with rapid aneurysmal expansion, malignancy, rupture-related changes, and mycotic aneurysm should be included in the differential diagnosis. Furthermore, when such an etiology is suspected, an operative strategy that facilitates histopathological confirmation may be warranted.

## Conclusion

We encountered a rare case of a ruptured DFAA caused by direct infiltration of DLBCL. Malignant lymphoma should be considered when patients present with rapidly enlarging or atypical peripheral aneurysms. Histological confirmation should be conducted whenever feasible.

## Declarations

### Data availability

The data supporting the findings of this case report are available from the corresponding author upon reasonable request.
